# Is Training With Gym Machines Safe After Hip Arthroplasty?—An *In Vivo* Load Investigation

**DOI:** 10.3389/fbioe.2022.857682

**Published:** 2022-03-24

**Authors:** Henryk Haffer, Alwina Bender, Alexander Krump, Sebastian Hardt, Tobias Winkler, Philipp Damm

**Affiliations:** ^1^ Center for Musculoskeletal Surgery Charité-Universitätsmedizin Berlin, Corporate Member of Freie Universität Berlin, Humboldt-Universität zu Berlin, Berlin, Germany; ^2^ Julius Wolff Institute, Charité—Universitätsmedizin Berlin, Corporate Member of Freie Universität Berlin, Humboldt-Universität zu Berlin and Berlin Institute of Health, Berlin, Germany; ^3^ Berlin Institute of Health Center for Regenerative Therapies, Charité—Universitätsmedizin Berlin, Corporate Member of Freie Universität Berlin, Humboldt-Universität zu Berlin, and Berlin Institute of Health, Berlin, Germany

**Keywords:** hip replacement, rehabilitation, sports, instrumented implants, leg extension, leg flexion, rope pull, leg press

## Abstract

**Background:** Training with gym machines is one of the most popular physical activities after total hip arthroplasty (THA). However, to date, there are no evidence-based recommendations for physical activity after THA, worldwide. The aim of the study is to evaluate the *in vivo* hip joint loads during exercises on four widely used gym machines in order to provide a source for an evidence-based patient counselling for arthroplasty surgeons.

**Methods:** The *in vivo* hip joint loads in seven patients (59.6 ± 6.4 years, 28.6 ± 2.1 kg/m^2^) with instrumented hip implants were assessed. The resulting force (F_res_), bending moment (M_bend_), and torsional moment (M_tors_) were evaluated during the training on leg curl/leg extension machines (loads: 20, 30, and 40 kg), leg press machine [backrest: 10°, 30°, and 60°; load: 50, 75, and 100%BW (bodyweight)], and a rope pull machine (abduction/adduction/flexion/extension; each ipsi- and contralateral; load 10 kg). These loads were compared with the loads during walking on treadmill at 4 km/h (median peak values: F_res_ 303%BW, M_bend_ 4.25%BWm, and M_tors_ 2.70%BWm).

**Results:** In each of the four performed exercises with a total of 23 different load conditions or exercise modes analyzed, a significantly lower or not different load was detected with respect to F_res_, M_bend_, and M_tors_ measured while walking with 4 km/h. Nevertheless, F_res_ and M_bend_ demonstrated a trend to increased loading during the ipsilateral monopod standing rope pull exercises hip flexion, extension, and abduction.

**Conclusion:** Based on our investigation, we assume that the investigated gym machines and external loads can be considered mainly as low-impact sports (with some exceptions) and thus as safe physical activity after THA. Due to the fact that the examinations were conducted in the mean 17.4 months after THA, the applicability of the results to the immediate postoperative period is limited.

## Introduction

Hip replacement is performed millions of times worldwide with increasing frequency and has already been described as the operation of the century (Kurtz et al., 2007; Learmonth et al., 2007; Pilz et al., 2018; Germany FSBo, 2020). Within the rising number of patients who have undergone total hip arthroplasty (THA), the increasing number of young patients under the age of 65 years is particularly noteworthy (Pabinger and Geissler, 2014). Accordingly, patients’ demands on the function of the replaced hip joint have considerably risen as well (Meek et al., 2020). The expectation of a timely return to work and physical activity after THA are of particular concern (Hoorntje et al., 2018). The raised ambitions in terms of the activity level are supported by a study of Innmann et al., which reported a constant level of physical activity in a 10-year follow-up, whereas Hara et al. even detected an increased physical activity after THA (Innmann et al., 2016; Hara et al., 2018). Despite the high expectations of the patients, the influence of physical activity on the THA outcome is still a subject of scientific discourse. There is evidence to suggest that moderate physical activity promotes bone metabolism contributing to improved osteointegration (Vogel et al., 2011). At the same time, torsional moments are suspected to affect the stability of the stem, leading to an increased risk of aseptic loosening (Bergmann et al., 1995; Bergmann et al., 2001; Gallo et al., 2010; Schmitt-Sody et al., 2011). The resultant force (F_res_) is considered as the main affecting force in the direction of the common load direction from the acetabulum to the femur head. The combination of force (F_res_) and bending and torsional moments in the biomechanical analysis is considered to represent the *in vivo* hip loads as close to reality as achievable. One study demonstrated an elevated revision rate in THA patients with an increased level of activity (Ollivier et al., 2012). Following this, the prevention of excessive wear and aseptic loosening might involve the obviation of high-impact sports (Berry and Bozic, 2010; Cherian et al., 2015). However, a relation between increased levels of activity and early THA failure revealed no conclusive evidence (Jassim et al., 2014).

Muscle strengthening is an indispensable part after THA aiming for stability and harmonious gait patterns and is also recognized to be associated with high patient satisfaction (Di Monaco et al., 2009; Di Monaco and Castiglioni, 2013). Instructed rehabilitation training with gym machines is widely established for THA patients in the postoperative schedule (Claes et al., 2012). Structured and standardized programs including the use of gym machines for muscle strengthening have proven their effectiveness after THA (Trudelle-Jackson and Smith, 2004). Training with gym machines not only is used in rehabilitation programs, but also gained increasing popularity as a leisure activity. Visiting a fitness center and training with gym machines is one of the most popular sports worldwide (Gough, 2021).

Despite the wide distribution and the frequent use of gym machines, the recommendations for sports after hip replacement remain with almost no evidence (Abe et al., 2014; Hoorntje et al., 2018). Due to this lack of evidence, there are still no conclusive evidence-based guidelines for sports after hip arthroplasty from the professional associations in orthopedics (Vogel et al., 2011; Meek et al., 2020; Vu-Han et al., 2020). Results from a survey among arthroplasty surgeons consider the use of gym machines as adequate for training after hip replacement (Vu-Han et al., 2020). However, although training exercises with gym machines are often performed as an activity in rehabilitative treatment programs and as sports for leisure, the effective *in vivo* loads on the prosthetic hip joint are still unknown (Claes et al., 2012). Consequently, this study aims to provide a source for evidence-based recommendations concerning the training with gym machines after hip replacement. We hypothesized that the measured *in vivo* loads for the various gym exercises would not be higher than for treadmill walking.

## Methods

### Ethics Statement

**FIGURE 1:**
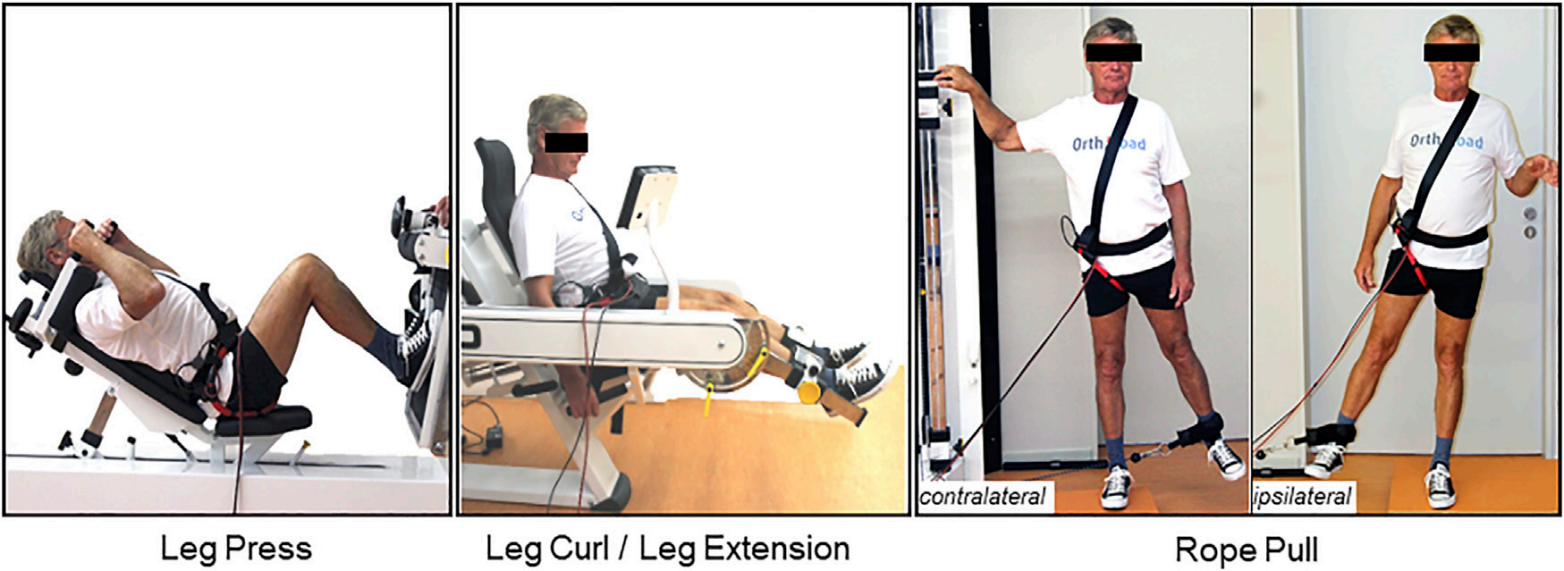
Depicting the selected gym machines: leg press machine, leg curl and leg extension machine, and ipsilateral and contralateral exercises on the rope pull machine (from left to right)**.** Ipsilateral and contralateral regarding the implanted instrumented hip prothesis, respectively. Rope pull exercises performed with ipsilateral side, standing on the contralateral leg referred to as ipsilateral. Rope pull exercises performed with contralateral side, standing on the ipsilateral leg referred to as contralateral.

The study was authorized by the Institutional Ethics Committee of Charité—Universitätsmedizin Berlin (EA2/057/09) and registered at the ‘“German Clinical Trials Register” (DRKS00000563). All investigations were performed in compliance with the applicable legal requirements. All patients gave written informed consent prior to participation in this study, in which they agreed to the implantation of the instrumented implants, *in vivo* load measurements and the publication of their images. Written informed consent was obtained from the individuals for the publication of any potentially identifiable images or data included in this article (Figure 1). It was not possible to involve patients or the public in the design, or conduct, or reporting, or dissemination plans of our research.

### Instrumented Hip Prosthesis

For the measurements of the *in vivo* joint load, an instrumented implant was used, which is capable of telemetrically transferring *in vivo* data. The technical details and the external equipment were described elsewhere (Bergmann et al., 2007; Graichen et al., 2007; Bergmann et al., 2008; Damm et al., 2010). The instrumented implant consists of a titanium alloy stem (TiAl_6_V_4_) and a 32-mm ceramic head (Al_2_O_3_) combined with a highly cross-linked polyethylene (XPE) inlay and a metallic pressfit cup (Ti_6_Al_4_V, Durasul, ZimmerBiomet). All patients were operated using a direct lateral approach. With the instrumented implants, six load components (three forces and three moments) can be measured *in vivo* with an accuracy of 1%–2%. The *in vivo* measured loads are transformed from the implant-based coordinate system into a femur-based coordinate system, fixed in the implant head center of a right-sided implant (Wu et al., 2002). If the implant is in the left leg, the loads are mirrored on to the right hip. The positive force components act in lateral, anterior, and superior directions in accordance with Wu et al. (2002).

### *In Vivo* Hip Joint Loads

**FIGURE 2:**
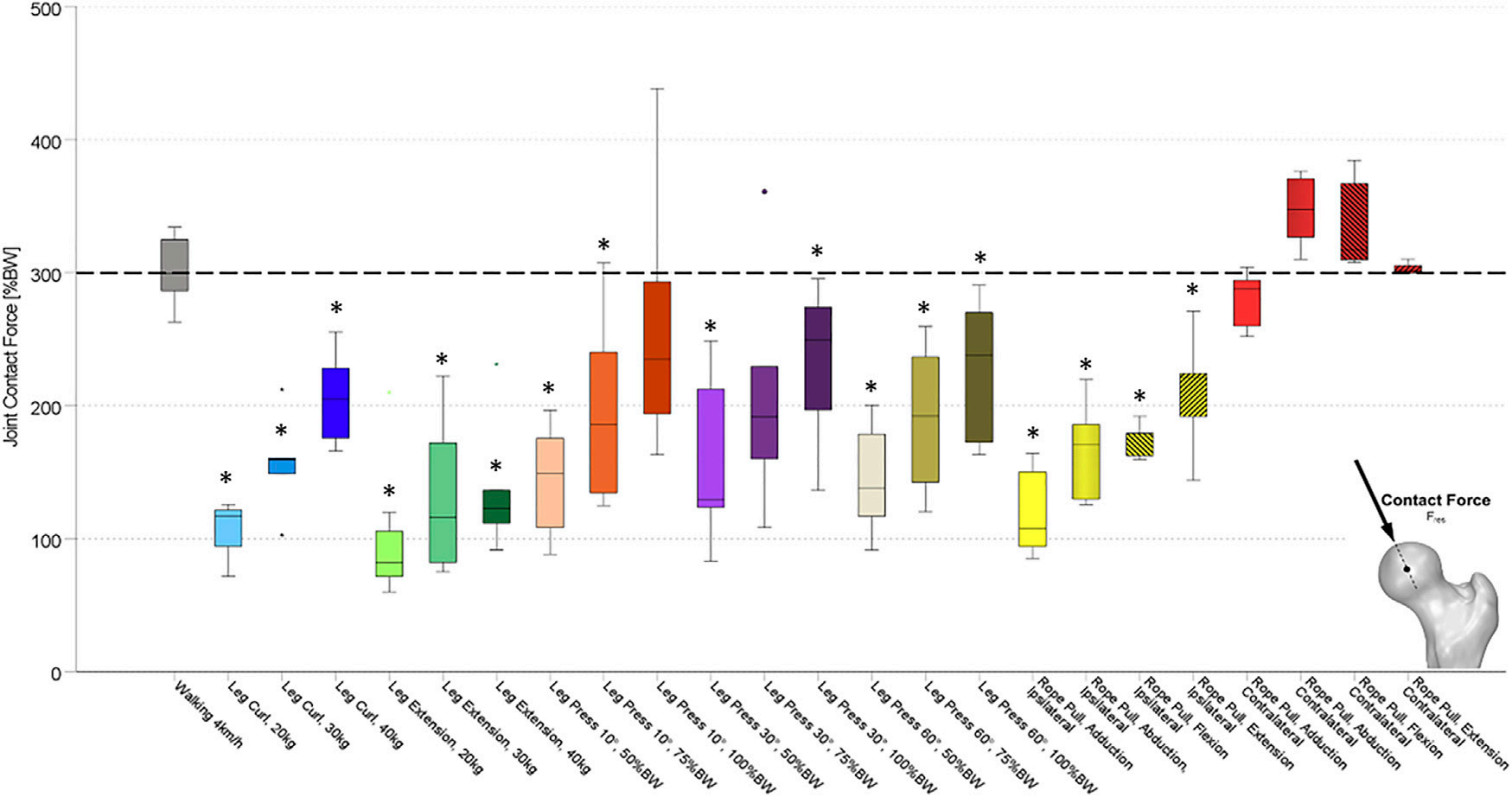
Resultant *in vivo* measured hip joint contact forces (F_res_) while performing the described activities on different training devices. Values were compared to walking as reference activity at a significance level of *p* ≤ 0.05. Significant differences to the values of walking are marked with an asterisk (*). Exercises were performed at the leg curl and the leg extension machine with different load conditions (20, 30, and 40 kg), at the leg press machine with different load conditions (50, 75, and 100%BW) and a different inclination of the backrest (10°, 30°, and 60°), and the rope pull machine with a load of 10 kg and hip adduction, abduction, flexion, and extension on ipsilateral and contralateral leg. Ipsilateral and contralateral regarding the standing leg is either the implanted instrumented hip prothesis (ipsilateral) or the not operated side (contralateral). Rope pull exercises performed with ipsilateral side, standing on the contralateral leg referred to as ipsilateral. Rope pull exercises performed with contralateral side, standing on the ipsilateral leg referred to as contralateral.

**FIGURE 3:**
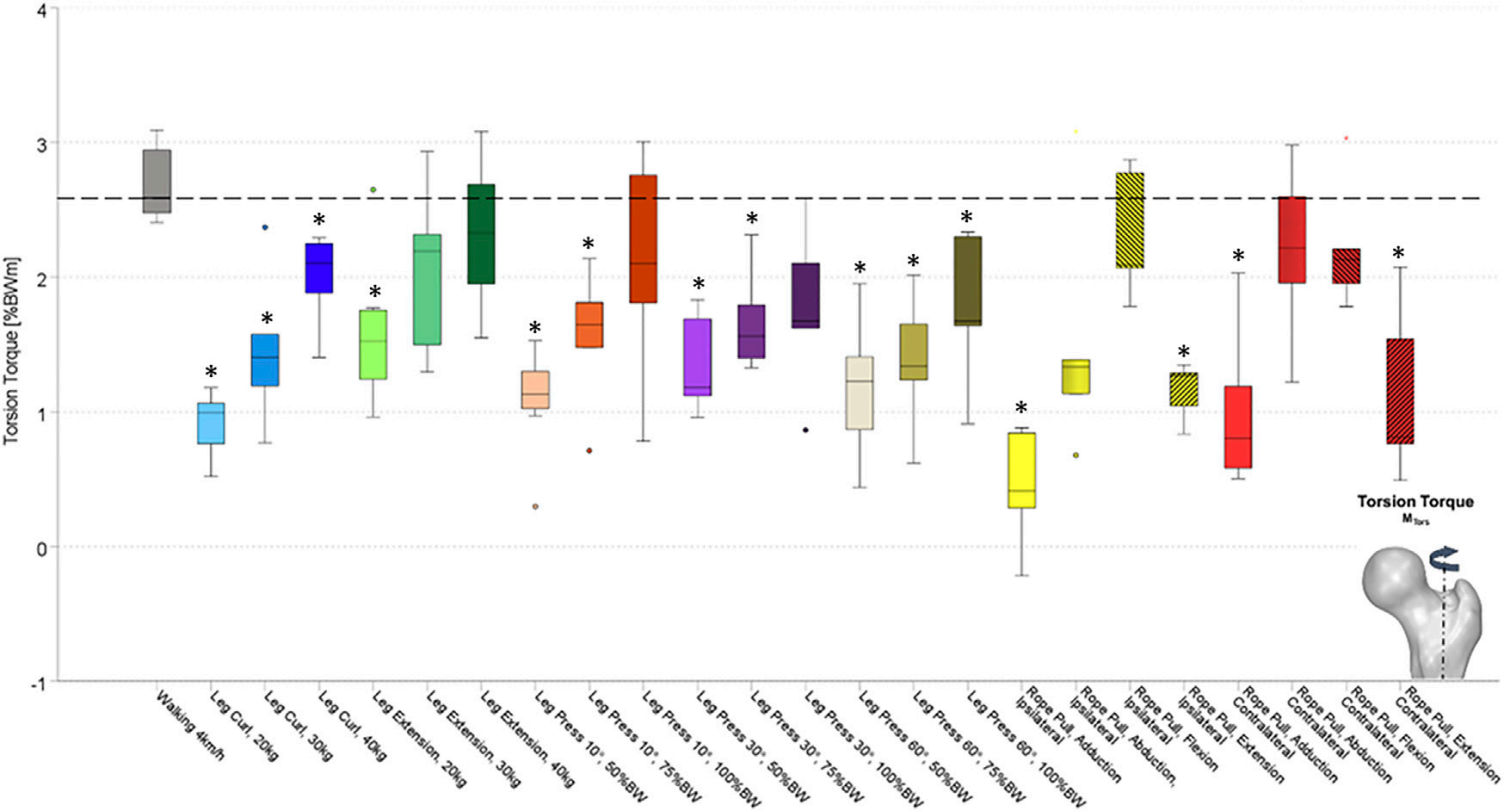
Resultant bending moments acting at the femoral neck (M_bend_), while performing several activities on different training devices. Values were compared to walking as reference activity at a significance level of *p* ≤ 0.05. Significant differences to walking are marked with an asterisk (*). Exercises were performed at the leg curl and the leg extension machine with different load conditions (20, 30, and 40 kg), at the leg press machine with different load conditions (50, 75, and 100%BW) and a different inclination of the backrest (10°, 30°, and 60°), and the rope pull machine with a load of 10 kg and hip adduction, abduction, flexion, and extension on ipsilateral and contralateral leg. Ipsilateral and contralateral regarding the standing leg is either the implanted instrumented hip prothesis (ipsilateral) or the not operated side (contralateral). Rope pull exercises performed with ipsilateral side, standing on the contralateral leg referred to as ipsilateral. Rope pull exercises performed with contralateral side, standing on the ipsilateral leg referred to as contralateral.

**FIGURE 4:**
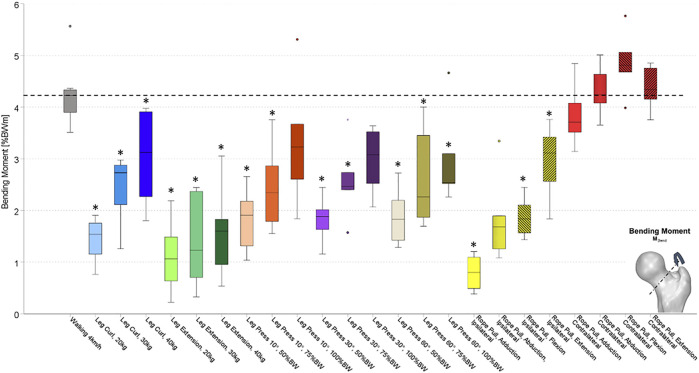
Resultant torsion torques acting on the femoral stem (M_tors_) while performing several activities on different training devices. Values were compared to walking as reference activity at a significance level of *p* ≤ 0.05. Significant differences to walking are marked with an asterisk (*). Exercises were performed at the leg curl and the leg extension machine with different load conditions (20, 30, and 40 kg), at the leg press machine with different load conditions (50, 75, and 100%BW) and a different inclination of the backrest (10°, 30°, and 60°), and the rope pull machine with a load of 10 kg and hip adduction, abduction, flexion, and extension on ipsilateral and contralateral leg. Ipsilateral and contralateral regarding the standing leg is either the implanted instrumented hip prothesis (ipsilateral) or the not operated side (contralateral). Rope pull exercises performed with ipsilateral side, standing on the contralateral leg referred to as ipsilateral. Rope pull exercises performed with contralateral side, standing on the ipsilateral leg referred to as contralateral.

All forces (F_res_) and moments (M_bend_, M_tors_) were normalized to the individual patient’s body weight (%BW) and %BWm (% body weight meter), respectively. The resultant joint contact force (F_res_) (Figure 2) was calculated by the three *in vivo* measured contact forces. Furthermore, from the three force components, the individual implant geometry, and the resulting lever arms, the torque around the femoral stem (M_tors_) (Figure 4) and the resultant bending moment (M_bend_) acting in the middle of the femoral neck (Figure 3) were determined.

### Participants and Measurements

Seven patients with osteoarthritis of the hip with such instrumented implants were included in the study (Table 1). They performed different load conditions at four different gym machines (Figure 1) with a minimum of eight repetitions, under the guidance of an experienced physiotherapist. Before performing the exercise, the physiotherapist demonstrated them to the patients and answered questions. The participants were adequately warmed up before the exercises and performed several trial exercises under guidance. Afterwards, the patient performed a minimum of eight self-controlled repetitions. In the analysis, the mean values of these repetitions of each individual exercise and load condition were considered. Furthermore, the *in vivo* acting joint loads were measured during walking on a treadmill with 4 km/h. Multiple gait cycles (30 gait cycles per individual participant) were considered for the analysis. Selected exercises of each measurement were published and can be downloaded at the public *in vivo* load database www.OrthoLoad.com.

**TABLE 1:** Patient characteristics; M, Mean; SD, standard deviation; BMI, body mass index at the time of measurement; THA, total hip arthroplasty, age (years) at time of measurement, weight (N) at the time of measurement.

Participants	Gender	Age [years]	Weight [N]	Height [cm]	BMI [kg/m^2^]	Time since THA [months]	Implant side
H2R	Male	63	774	172	26.7	25	Right
H3L	Male	61	896	168	32.4	24	Left
H4L	Male	52	828	178	26.6	23	Left
H5L	Female	64	855	168	29.8	19	Left
H6R	Male	69	841	176	28.1	12	Right
H7R	Male	53	924	179	29.3	12	Right
H8L	Male	55	841	178	27.1	7	Left
M ±SD	—	59.6 ± 6.4	851.3 ± 48.4	174.1 ± 4.8	28.6 ± 2.1	17.4 ± 7.1	—

### Gym Machines

#### Leg Press

On the leg press, the whole body has to move in the horizontal plane, while the movements were performed with three different inclination angles of the backrest (10°, 30°, and 60°) and three external load conditions (50, 75, and 100%BW). The load as a function of body weight (%BW) for the leg press exercise was chosen, because the movement is comparable to squats and the effective loads performing this movement are dependent on the body weight. The movement was started with 90° flexion at the knee and hip joint. The extension phase was stopped shortly before straight leg position and the patients went back to the start position from there.

#### Leg Curl/Leg Extension

At the leg curl/leg extension machine, the subjects sat in an upright position. They performed with both legs a knee flexion from 0° to 90° and a knee extension from 90° to 0° with a loading condition of 20, 30, and 40 kg.

#### Rope Pull

The subjects performed separately an abduction/adduction and a flexion/extension of the hip joint with a load of 10 kg with a straight leg. The movements were executed with the ipsilateral and also with the contralateral leg.

### Data Analysis

The four different exercises with the differing loads and variations were each compared against the reference activity of walking at 4 km/h (Table 2; Figures 2–4) and additionally compared between different load conditions in the same exercise (described in the results section). All data displayed refer to the median results obtained. The median results were assessed by the average of multiple trials for each patient individually, and from these intra-individual medians, the median results were calculated over all participants. SPSS (IBM, Armonk, NY, United States) was used for the statistical evaluation. The Wilcoxon signed rank test was applied and the level of statistical significance was set at *p* < 0.05.

**TABLE 2:** Reference activity walking versus each investigated activity depicting the resultant force F_res_, bending moment M_bend_, and torsion torque M_tors_ with the results given as delta ∆ (%) of the median peak values in relation to walking, bold—*in vivo* measured peak values are significantly smaller relative to walking *in vivo* measured peak values.

Activity	F_res_		M_bend_		M_tors_	
	Δ (%)	*p*-value	Δ (%)	*p*-value	Δ (%)	*p*-value
Leg curl 20 kg	−65	0.018	−66	0.018	−67	0.018
Leg curl 30 kg	−48	0.028	−42	0.028	−46	0.028
Leg curl 40 kg	−32	0.028	−27	0.028	−25	0.028
Leg extension 20 kg	−66	0.018	−74	0.018	−41	0.028
Leg extension 30 kg	−57	0.028	−67	0.028	−23	0.173
Leg extension 40 kg	−55	0.028	−62	0.028	−14	0.345
Leg press, backrest 10°, 50%BW	−53	0.018	−58	0.018	−60	0.018
Leg press, backrest 10°, 75%BW	−35	0.028	−43	0.028	−42	0.028
Leg press, backrest 10°, 100%BW	−14	0.141	−22	0.116	−23	0.116
Leg press, backrest 30°, 50%BW	−47	0.043	−57	0.043	−50	0.043
Leg press, backrest 30°, 75%BW	−31	0.080	−39	0.043	−38	0.043
Leg press, backrest 30°, 100%BW	−24	0.043	−30	0.080	−35	0.080
Leg press, backrest 60°, 50%BW	−51	0.028	−56	0.018	−57	0.018
Leg press, backrest 60°, 75%BW	−37	0.028	−39	0.028	−49	0.028
Leg press, backrest 60°, 100%BW	−25	0.043	−29	0.043	−35	0.043
Rope pull, ipsilateral performed, adduction	−58	0.018	−78	0.018	−85	0.018
Rope pull, ipsilateral performed, abduction	−37	0.028	−37	0.075	−19	0.345
Rope pull, ipsilateral performed, flexion	−39	0.018	−51	0.018	−6	0.612
Rope pull, ipsilateral performed, extension	−30	0.018	−29	0.018	−57	0.018
Rope pull, contralateral performed, adduction	−7	0.075	−6	0.116	−68	0.028
Rope pull, contralateral performed, abduction	+14	0.075	+9	0.463	−22	0.249
Rope pull, contralateral performed, flexion	+10	0.249	+25	0.173	−13	0.249
Rope pull, contralateral performed, extension	+3	0.463	+9	0.600	−50	0.028

Wilcoxon signed rank test (two-sided) was used to determine significant differences from the various activities to the reference activity walking. Ipsilateral and contralateral regarding the standing leg does indicate either the implanted instrumented hip prothesis (ipsilateral) or the not operated side (contralateral). Rope pull exercises performed with ipsilateral side, standing on the contralateral leg, referred to as ipsilateral. Rope pull exercises performed with contralateral side, standing on the ipsilateral leg, referred to as contralateral.

## Results

During treadmill walking, median peak values of F_res_ up to 303%BW were measured *in vivo* acting at the hip joint. Furthermore, corresponding median peak values for M_bend_ of 4.25%BWm and for M_tors_ of 2.70%BWm were measured (dotted lines in Figures 2–4).

### Leg Curl

During the training at the leg curl machine using loads of 20, 30, and 40 kg, peak values of F_res_ between 107 and 207%BW were measured (Figure 2). Median peak values for M_bend_ ranged from 1.44 to 3.06%BWm (Figure 3) and those for M_tors_ were between 0.9 and 2.03%BWm (Figure 4). Significantly smaller median peak values of F_res_, M_bend_, and M_tors_ were observed during leg curl exercises in all three loads performed compared to the reference activity walking (Table 2). An increase of the load from 20 to 30 kg significantly enhanced the F_res_ by 40%, M_tors_ by 52%, and M_bend_ by 71%, and an increase from 20 to 40 kg led to a significantly raised F_res_ by 78%, M_tors_ by 140%, and M_bend_ by 95%.

### Leg Extension

When the subjects used the leg extension machine with the loads 20, 30, and 40 kg, median peak values of F_res_ between 102 and 136%BW were measured (Figure 2). The hip joint was loaded by M_bend_ between 1.11 and 1.60%BWm (Figure 3) and M_tors_ between 1.60 and 2.33%BWm (Figure 4). In all three load conditions during leg extension exercises, significantly lower median peak values of F_res_, M_bend_, and M_tors_ compared to the reference activity walking were demonstrated, except for M_tors_ with the loads 30 and 40 kg (Table 2). An increase of the machine load from 20 to 30 kg and from 20 to 40 kg was followed by an increase of F_res_ and M_tors_ by 33% and M_bend_ by 31% and of F_res_ by 38%, M_tors_ by 40%, and M_bend_ by 56%, respectively.

### Leg Press

By using the Leg Press machine, three different load conditions (50/75/100%BW) of the machine at three different backrest positions (10°/30°/60°) were investigated. The median peak values for F_res_, M_bend_, and M_tors_ in all backrest positions and load conditions were significantly smaller than during walking. Only some load conditions at the leg press machine revealed no differences compared to the reference activity walking (100%BW load condition, 10° backrest position: F_res_, M_bend_, and M_tors_; 100%BW load condition, 30° backrest position: M_bend_ and M_tors_; 75%BW load condition, 30° backrest position: F_res_). (Table 2).

During the various modes of the leg press exercises, median peak values of F_res_ between 143 and 260%BW (10° backrest), 168 and 230%BW (30° backrest), and 147 and 227%BW (60° backrest) were measured (Figure 2). An increase of the machine load from 50 to 75%BW was followed by an increase of F_res_ by 39% (10°), 48% (30°), and 60% (60°). However, only the changes from 50 to 100%BW at 10° (+77%), from 50 to 75%BW at 30° (+48%), and in all conditions at 60° backrest position (50%BW to 75%BW: +60%; 50%BW to 100%BW: +51%) were significant.

The corresponding median peak values of M_bend_ increased with enhanced external loads ranging from 1.80 to 3.31%BWm (10°), from 1.82 to 3.0%BWm (30°), and from 1.86 to 3.01%BWm (60°) (Figure 3). An increase of the loading condition from 50 to 75%BW led to an enhancement of M_bend_ of 24% (10°), 31% (30°), and 25% (60°). Only the changes at the 10° backrest position were significant when the machine load increased from 50 to 100%BW (+37%) and from 75 to 100%BW (+70%).

Median peak values of M_tors_ (Figure 4) were measured between 1.09 and 2.09%BWm (10°), between 1.36 and 1.77%BWm (30°), and between 1.17 and 1.76%BWm (60°). When the external load was increased from 50 to 75%BW, M_tors_ was increased by 44% (10°), 24% (30°), and 17% (60°). However, by a further increase of the machine load from 75%BW to 100%BW, M_tors_ increased by 33% (10°), 5% (30°), and 29% (60°). Only the changes of M_tors_, followed by an increase from 50 to 75%BW and from75 to 100%BW, with a 10° backrest position, were significantly different between the two load conditions.

### Rope Pull

At the rope pull machine, hip abduction, adduction, extension, and flexion with 10 kg machine load were performed, standing on each of the ipsilateral (implanted) and the contralateral leg. It is referred to the following as ipsilateral when the exercise was performed with the ipsilateral leg and standing on contralateral leg, and *vice versa*.

The median peak values for F_res_, M_bend_, and M_tors_ in all four exercises performed with the ipsilateral leg (standing on the contralateral leg) were significantly lower than for walking or did not differ for M_tors_ flexion and abduction exercise and M_bend_ (abduction). The median peak values for F_res_, M_bend_, and M_tors_ in all four exercises performed with the contralateral leg (standing on the ipsilateral leg) were significantly lower to walking, with the exception of F_res_ and M_bend_ for the abduction, flexion, and extension exercise with an increase compared to walking.

Median peak values for F_res_ performing with the contralateral leg ranged from 282%BW (adduction) to 343%BW (abduction), while performing with the ipsilateral leg resulted in values for F_res_ between 128%BW (adduction) and 212%BW (extension). All exercises on the rope pull machine performed with the ipsilateral leg displayed distinct smaller median peak values for F_res_ with a decrease between 28% (extension) and 63% (adduction) compared to the contralateral leg.

Contralaterally performed exercises led to M_bend_ median peak values between 4.0%BWm (adduction) and 5.33%BWm (flexion) compared to the ipsilateral leg between 0.93%BWm (adduction) and 3.02%BWm (extension). All exercises demonstrated distinct decreases when performed with the ipsilateral leg (standing on the contralateral leg) compared to the contralateral leg with decreases ranging from 30% (extension) to 78% (adduction).

Median peak values for M_tors_ were measured between 1.02%BWm (adduction) and 2.22%BWm (flexion) when performed with the contralateral leg, compared to the ipsilateral leg ranging from 0.44%BWm (adduction) to 2.32%BWm (flexion). Performance with the ipsilateral leg (standing on the contralateral leg) compared to contralateral performance (standing on the ipsilateral leg) led to smaller median peak values for M_tors_ with a decrease between 31% (abduction) and 57% (adduction) and increases of 2% (extension) and 5% (flexion) with none of the comparisons between ipsilateral and contralateral reached statistical significance.

## Discussion

The study detected in each of the four performed exercises (leg curl, leg extension, leg press, and rope pull) with a total of 23 different load conditions or exercise modes analyzed a significantly lower or a non-differing load with respect to F_res_, M_tors_, and M_bend_ compared to the reference activity level walking.

There is scientific consensus that unrestricted level walking after primary THA is safe for the patients (Swanson et al., 2009). Therefore, we have chosen the *in vivo* resultant joint contact force as well as the bending moment at the femur neck and torsional torque at the femur stem, occurring during level walking as a reference, to compare them with the *in vivo* loads during the different exercises on the gym machines. The F_res_, M_tors_, and M_bend_ determined during level walking were comparable to the values measured in previously reported *in vivo* load investigations (Bergmann et al., 2001; Damm et al., 2013; Damm et al., 2015; Damm et al., 2017).

It is necessary to differentiate between two applications of the use of gym machines. On the one hand, exercises on gym machines are applied in the context of rehabilitative programs after THA, under the supervision of physiotherapists. On the other hand, the muscle strengthening exercises performed on gym machines in fitness centers are one of the most popular leisure activities for people all over the world.

In the immediate postoperative period, device-supported training of the muscles surrounding the hip is not recommended. In this phase, during the first postoperative week, the mobilization of the patient and passively assisted movement of the hip joint are in the focus of attention. In the following post-primary phase, a rehabilitation program under supervised physiotherapy is often performed to improve mobilization, coordination, stretching, and strengthening of the hip joint encompassing muscles. From the 4th to 5th postoperative week after THA, a device-supported postoperative physiotherapy may be started (including the exercises on the gym machines we have investigated), individually adapted to the patient’s abilities (Claes et al., 2012).

We would like to point out that strengthening abduction exercises with resistance or on gym machines immediately after THA are not advisable. On the one hand, this is to protect the osteointegration of the cementless implant from micromotions at the stem–bone interface (Hofmann et al., 1997; Liu et al., 2020). Increased torsional moments can endanger osteointegration and thus prosthesis stability (Viceconti et al., 2000; Chen et al., 2014). On the other hand, abduction training with resistance in the immediate phase after THA *via* lateral and anterolateral approaches might increase the risk of THA dislocations and should therefore be avoided (Rope pull abduction exercise). However, focused training of the gluteus musculature is crucial. It is known that hip abductors are essential for a balanced gait pattern and activities of the daily life (Mickelborough et al., 2004; Tirosh and Sparrow, 2005). Nevertheless, musculature imbalances in the gluteal region frequently occur after hip replacement (Müller et al., 2011). This can also be followed by critically increased joint loads (Damm et al., 2018). It was reported that specific training of the hip abductors in rehabilitative programs improves the clinical outcome and patient satisfaction (Unlu et al., 2007; Jacobs et al., 2009; Benedetti et al., 2021).

The leg curl, leg extension, and leg press exercises revealed significantly lower and rarely no differences in the resultant force and bending and torsional moments affecting the hip prothesis compared to the reference activity walking. However, the anticipated increase of F_res_, M_tors_, and M_bend_ with increasing load conditions is evident, without exceeding the values of walking. It should be noted, however, that when comparing the individual median values of the individual participants to their individual median walking value, increases above the individual median walking level of the participant occurred for some exercises (Supplementary Tables 1–3). Interestingly, the various backrest positions (10°/30°/60°) on the leg press machine had no relevant influence on the acting forces and moments on the hip joint. The aforementioned exercises were performed with both legs; we assume distinctly higher *in vivo* loads for the single-legged exercise. Moreover, it has been illustrated that an increase in external load does not necessarily lead to an equally large increase in load *in vivo*. This is an essential finding, and in consequence, it must be mentioned that a simple linear estimation of the *in vivo* acting loads according to the external load applied is not feasible. A possible explanation is, to overpower the increase of the externally applied loading, the individual muscle activation and muscle balancing are changing in a non-optimal biomechanical way. However, due to these modified muscle balancing and muscle recruitment strategies, the internal joint loads can differentiate than it is expected from the external load increase. Since we have not performed electromyography, we cannot prove the hypothesis. Another plausible hypothesis is an increased load on the contralateral leg due to pain or fear of movement.

In the rope pull machine exercises, it is necessary to distinguish between the exercises performed with the ipsilateral leg (standing on the contralateral leg) and with the contralateral leg (standing on the ipsilateral). F_res_, M_tors_, and M_bend_ for the ipsilaterally performed exercises were significantly lower than those for walking. This was not observed for the contralaterally performed rope pull exercises (standing on the ipsilateral leg). Here, F_res_ and M_bend_ revealed an increase in hip abduction, flexion, and extension exercise. Overall, F_res_ and M_bend_ during single-leg stance were distinctly higher in the exercises performed with the contralateral leg compared to the rope pull exercises performed ipsilaterally (standing on the instrumented hip prosthesis, named ipsilateral). This indicates the exceptional load on the prosthesis during monopod standing and has been reported in other studies (Haffer et al., 2021). The flexion and abduction movement with the rope pull machine with the contralateral leg increases the lever arm. The ipsilateral abductor musculature is compensating for this effect, leading to the observed increase of F_res_ and M_bend_. Since the increase here was not substantial compared to the reference walking, we assume that there was no relevant loading during these exercises. However, attention should be paid to the accurate exercise performance, initial execution under supervision of a physiotherapist might be considered, and external assistance to balance the patients may be applied.

To date, there are no evidence-based guidelines from the orthopedic professional associations on which types of sports are recommended after THA, apart from the advice to avoid so-called high-impact sports that are not specified by consensus (Vogel et al., 2011). It is assumed that the currently used implants and fixation techniques are adequate for amateur sports level (Jassim et al., 2014). Besides, there are only a few studies investigating on *in vivo* loads in physiotherapy, aquatic exercises, and Nordic Walking (Schwachmeyer et al., 2013; Kutzner et al., 2017; Palmowski et al., 2021). There is no evidence in the short- and midterm follow-up that increased implant failure occurs with increased sports activity (Meek et al., 2020). Therefore, the responsibility for the decision-making process remains with the arthroplasty surgeon and the THA patient. The decision for each patient should be individualized and based on the previous athletic experience, health status, bone condition, and risk tolerance, as well as the possible consequences of increased wear and aseptic loosening (Meira and Zeni, 2014; Hoorntje et al., 2018).

Several limitations of our investigation need to be considered. One should keep in mind the limited but worldwide unique study population. Especially when extrapolating the results to a general THA patient population, caution is advised. In interpreting the results, we would like to point out that the study population is homogeneous in specific characteristics (younger, active patients) and therefore conclusions for patients with considerably different characteristics are only possible to a limited extent. It cannot be entirely ruled out that the measured *in vivo* loads of an individual subject in a single exercise or load condition may significantly exceed the individual subject’s reference activity level walking. The use of external loads with body weight-adjusted (%BW) or absolute values (kg) at different gym machine exercises may have influenced the outcomes. Some exercises or load conditions were not performed by all participants, and this may have led to bias (Supplementary Tables 1–3). It should be considered that the examinations were conducted in the mean 17.4 months after THA. Therefore, the applicability of the results to the immediate postoperative period is limited. It is reported that the direct lateral approach used in our study leads to a reduction in muscle volume and fatty degeneration, which may have a possible influence on the *in vivo* hip joint loads in a short-term follow-up (Damm et al., 2018; Damm et al., 2019). Since we present results of a midterm follow-up (17.4 months after THA), we do not assume a decisive impact of the direct lateral approach on the joint loads. Nevertheless, in one subject, the *in vivo* hip joint loads were measured 7 months after lateral THA approach. A potential influence on the measurements cannot be entirely ruled out. Since we used a standard commercially available cementless prosthesis, no influence of the prosthesis on the *in vivo* loads is expected.

The study in this unique patient cohort with instrumented implants is the first to demonstrate the *in vivo* hip joint loads during the most common exercises on gym machines. In all four exercises (leg curl, leg extension, leg press, and rope pull) with a total of 23 different load levels or variations, we determined significantly lower or not differing loads compared to normal walking, except for an increase in F_res_ and M_Bend_ in monopod standing on the leg with the instrumented implant performing hip flexion, extension, and abduction on the rope pull machine. Thus, attention might be drawn to the possibly increased loads when performing this ipsilateral monopod standing exercise. Therefore, we recommend ensuring a supervised execution with a load condition individually adapted to the patients’ body weight and training condition and would refrain from performing monopod standing in the rehabilitative phase. According to our results, we assume that the investigated gym machines can be considered as low-impact sports with the previously mentioned constraints and thus as safe physical activity after THA.

## Data Availability

The datasets presented in this study can be found in online repositories. The names of the repository/repositories and accession number(s) can be found at: www.OrthoLoad.com.

## References

[B1] Abe H. Sakai T. Nishii T. Takao M. Nakamura N. Sugano N. (2014). Jogging after Total Hip Arthroplasty. Am. J. Sports Med. 42 (1), 131–137. 10.1177/0363546513506866 24114754

[B2] Benedetti M. G. Cavazzuti L. Amabile M. Tassinari E. Valente G. Zanotti G. (2021). Abductor Muscle Strengthening in THA Patients Operated with Minimally-Invasive Anterolateral Approach for Developmental Hip Dysplasia. HIP Int. 31 (1), 66–74. 10.1177/1120700019877174 31544524

[B3] Bergmann G. Graichen F. Rohlmann A. Westerhoff P. Heinlein B. Bender A. (2008). Design and Calibration of Load Sensing Orthopaedic Implants. J. Biomech. Eng. 130 (2), 021009. 10.1115/1.2898831 18412496

[B4] Bergmann G. Deuretzbacher G. Heller M. Graichen F. Rohlmann A. Strauss J. (2001). Hip Contact Forces and Gait Patterns from Routine Activities. J. Biomech. 34 (7), 859–871. 10.1016/s0021-9290(01)00040-9 11410170

[B5] Bergmann G. Graichen F. Rohlmann A. (1995). Is Staircase Walking a Risk for the Fixation of Hip Implants? J. Biomech. 28 (5), 535–553. 10.1016/0021-9290(94)00105-d 7775490

[B6] Bergmann G. Graichen F. Rohlmann A. Westerhoff P. Bender A. Gabel U. (2007). Die Belastung Orthopädischer Implantate. Orthopäde 36 (3), 195–204. 10.1007/s00132-007-1055-x 17333070

[B7] Berry D. J. Bozic K. J. (2010). Current Practice Patterns in Primary Hip and Knee Arthroplasty Among Members of the American Association of Hip and Knee Surgeons. J. Arthroplasty 25 (6 Suppl. l), 2–4. 10.1016/j.arth.2010.04.033 20580196

[B8] Chen W.-C. Lai Y.-S. Cheng C.-K. Chang T.-K. (2014). A Cementless, Proximally Fixed Anatomic Femoral Stem Induces High Micromotion with Nontraumatic Femoral Avascular Necrosis: A Finite Element Study. J. Orthopaedic Translation 2 (3), 149–156. 10.1016/j.jot.2014.03.002

[B9] Cherian J. J. Jauregui J. J. Banerjee S. Pierce T. Mont M. A. (2015). What Host Factors Affect Aseptic Loosening after THA and TKA? Clin. Orthop. Relat. Res. 473 (8), 2700–2709. 10.1007/s11999-015-4220-2 25716213 PMC4488212

[B10] Claes L. Krischner P. Perka C. Rudert M. (2012). AE-Manual der Endoprothetik Hüfte und Hüftrevision. Berlin/Heidelberg, Germany: Springer.

[B11] Damm P. Bender A. Bergmann G. (2015). Postoperative Changes in *In Vivo* Measured Friction in Total Hip Joint Prosthesis during Walking. PLoS One 10 (3), e0120438. 10.1371/journal.pone.0120438 25806805 PMC4373913

[B12] Damm P. Brackertz S. Streitparth F. Perka C. Bergmann G. Duda G. N. (2019). ESB Clinical Biomechanics Award 2018: Muscle Atrophy-Related Increased Joint Loading after Total Hip Arthroplasty and Their Postoperative Change from 3 to 50 Months. Clin. Biomech. 65, 105–109. 10.1016/j.clinbiomech.2019.04.008 31026763

[B13] Damm P. Graichen F. Rohlmann A. Bender A. Bergmann G. (2010). Total Hip Joint Prosthesis for *In Vivo* Measurement of Forces and Moments. Med. Eng. Phys. 32 (1), 95–100. 10.1016/j.medengphy.2009.10.003 19889565

[B14] Damm P. Kutzner I. Bergmann G. Rohlmann A. Schmidt H. (2017). Comparison of *In Vivo* Measured Loads in Knee, Hip and Spinal Implants during Level Walking. J. Biomech. 51, 128–132. 10.1016/j.jbiomech.2016.11.060 27914627

[B15] Damm P. Schwachmeyer V. Dymke J. Bender A. Bergmann G. (2013). *In Vivo* hip Joint Loads during Three Methods of Walking with Forearm Crutches. Clin. Biomech. 28 (5), 530–535. 10.1016/j.clinbiomech.2012.12.003 23643290

[B16] Damm P. Zonneveld J. Brackertz S. Streitparth F. Winkler T. (2018). Gluteal Muscle Damage Leads to Higher *In Vivo* Hip Joint Loads 3 Months after Total Hip Arthroplasty. PLoS One 13 (1), e0190626. 10.1371/journal.pone.0190626 29315350 PMC5760017

[B17] Di Monaco M. Castiglioni C. (2013). Which Type of Exercise Therapy Is Effective after Hip Arthroplasty? A Systematic Review of Randomized Controlled Trials. Eur. J. Phys. Rehabil. Med. 49 (6), 893–3. quiz 21-3. 24172644

[B18] Di Monaco M. Vallero F. Tappero R. Cavanna A. (2009). Rehabilitation after Total Hip Arthroplasty: a Systematic Review of Controlled Trials on Physical Exercise Programs. Eur. J. Phys. Rehabil. Med. 45 (3), 303–317. 19238130

[B19] Gallo J. Slouf M. Goodman S. B. (2010). The Relationship of Polyethylene Wear to Particle Size, Distribution, and Number: A Possible Factor Explaining the Risk of Osteolysis after Hip Arthroplasty. J. Biomed. Mater. Res. B Appl. Biomater. 94 (1), 171–177. 10.1002/jbm.b.31638 20524192

[B20] Germany FSBo (2020). The 20 Most Frequent Surgeries in Germany 2019. Available at: https://www.destatis.de/DE/Themen/Gesellschaft-Umwelt/Gesundheit/Krankenhaeuser/Tabellen/drg-operationen-insgesamt.html .

[B21] Gough C. (2021). Health & Fitness Clubs - Statistics & Facts: Statista. Available at: www.statista.com/topics/1141/health-and-fitness-clubs/#dossierKeyfigures .

[B22] Graichen F. Arnold R. Rohlmann A. Bergmann G. (2007). Implantable 9-channel Telemetry System for *In Vivo* Load Measurements with Orthopedic Implants. IEEE Trans. Biomed. Eng. 54 (2), 253–261. 10.1109/tbme.2006.886857 17278582

[B23] Haffer H. Popovic S. Martin F. Hardt S. Winkler T. Damm P. (2021). *In Vivo* loading on the Hip Joint in Patients with Total Hip Replacement Performing Gymnastics and Aerobics Exercises. Sci. Rep. 11 (1), 13395. 10.1038/s41598-021-92788-7 34183711 PMC8239021

[B24] Hara D. Hamai S. Komiyama K. Motomura G. Shiomoto K. Nakashima Y. (2018). Sports Participation in Patients after Total Hip Arthroplasty vs Periacetabular Osteotomy: A Propensity Score-Matched Asian Cohort Study. The J. Arthroplasty 33 (2), 423–430. 10.1016/j.arth.2017.08.035 28947372

[B25] Hofmann A. A. Bloebaum R. D. Bachus K. N. (1997). Progression of Human Bone Ingrowth into Porous-Coated Implants: Rate of Bone Ingrowth in Humans. Acta Orthopaedica Scand. 68 (2), 161–166. 10.3109/17453679709004000 9174454

[B26] Hoorntje A. Janssen K. Y. Bolder S. B. T. Koenraadt K. L. M. Daams J. G. Blankevoort L. (2018). The Effect of Total Hip Arthroplasty on Sports and Work Participation: A Systematic Review and Meta-Analysis. Sports Med. 48 (7), 1695–1726. 10.1007/s40279-018-0924-2 29691754 PMC5999146

[B27] Innmann M. M. Weiss S. Andreas F. Merle C. Streit M. R. (2016). Sports and Physical Activity after Cementless Total Hip Arthroplasty with a Minimum Follow-Up of 10 Years. Scand. J. Med. Sci. Sports 26 (5), 550–556. 10.1111/sms.12482 26041645

[B28] Jacobs C. A. Lewis M. Bolgla L. A. Christensen C. P. Nitz A. J. Uhl T. L. (2009). Electromyographic Analysis of Hip Abductor Exercises Performed by a Sample of Total Hip Arthroplasty Patients. J. Arthroplasty 24 (7), 1130–1136. 10.1016/j.arth.2008.06.034 18757169

[B29] Jassim S. S. Douglas S. L. Haddad F. S. (2014). Athletic Activity after Lower Limb Arthroplasty. Bone Jt. J. 96-b (7), 923–927. 10.1302/0301-620x.96b7.31585 24986946

[B30] Kurtz S. Ong K. Lau E. Mowat F. Halpern M. (2007). Projections of Primary and Revision Hip and Knee Arthroplasty in the United States from 2005 to 2030. J. Bone Jt. Surg. 89 (4), 780–785. 10.2106/jbjs.f.00222 17403800

[B31] Kutzner I. Richter A. Gordt K. Dymke J. Damm P. Duda G. N. (2017). Does Aquatic Exercise Reduce Hip and Knee Joint Loading? *In Vivo* Load Measurements with Instrumented Implants. PLoS One 12 (3), e0171972. 10.1371/journal.pone.0171972 28319145 PMC5358747

[B32] Learmonth I. D. Young C. Rorabeck C. (2007). The Operation of the century: Total Hip Replacement. The Lancet 370 (9597), 1508–1519. 10.1016/s0140-6736(07)60457-7 17964352

[B33] Liu Y. Rath B. Tingart M. Eschweiler J. (2020). Role of Implants Surface Modification in Osseointegration: A Systematic Review. J. Biomed. Mater. Res. 108 (3), 470–484. 10.1002/jbm.a.36829 31664764

[B34] Meek R. M. D. Treacy R. Manktelow A. Timperley J. A. Haddad F. S. (2020). Sport after Total Hip Arthroplasty: Undoubted Progress but Still Some Unknowns. Bone Jt. J. 102-b (6), 661–663. 10.1302/0301-620x.102b6.bjj-2020-0208 PMC7241060 32475237

[B35] Meira E. P. Zeni J. Jr. (2014). Sports Participation Following Total Hip Arthroplasty. Int. J. Sports Phys. Ther. 9 (6), 839–850. 25383251 PMC4223292

[B36] Mickelborough J. van der Linden M. L. Tallis R. C. Ennos A. R. (2004). Muscle Activity during Gait Initiation in normal Elderly People. Gait & Posture 19 (1), 50–57. 10.1016/s0966-6362(03)00016-x 14741303

[B37] Müller M. Tohtz S. Springer I. Dewey M. Perka C. (2011). Randomized Controlled Trial of Abductor Muscle Damage in Relation to the Surgical Approach for Primary Total Hip Replacement: Minimally Invasive Anterolateral versus Modified Direct Lateral Approach. Arch. Orthop. Trauma Surg. 131 (2), 179–189. 10.1007/s00402-010-1117-0 20490520

[B38] Ollivier M. Frey S. Parratte S. Flecher X. Argenson J.-N. (2012). Does Impact Sport Activity Influence Total Hip Arthroplasty Durability? Clin. Orthop. Relat. Res. 470 (11), 3060–3066. 10.1007/s11999-012-2362-z 22535588 PMC3462849

[B39] Pabinger C. Geissler A. (2014). Utilization Rates of Hip Arthroplasty in OECD Countries. Osteoarthritis and Cartilage 22 (6), 734–741. 10.1016/j.joca.2014.04.009 24780823

[B40] Palmowski Y. Popovic S. Schuster S. G. Hardt S. Damm P. (2021). *In Vivo* analysis of Hip Joint Loading on Nordic Walking Novices. J. Orthop. Surg. Res. 16 (1), 596. 10.1186/s13018-021-02741-7 34649562 PMC8515744

[B41] Pilz V. Hanstein T. Skripitz R. (2018). Projections of Primary Hip Arthroplasty in Germany until 2040. Acta Orthopaedica 89 (3), 308–313. 10.1080/17453674.2018.1446463 29504824 PMC6055773

[B42] Schmitt-Sody M. Pilger V. Gerdesmeyer L. (2011). Rehabilitation und Sport nach Hüfttotalendoprothese. Orthopäde 40 (6), 513–519. 10.1007/s00132-011-1761-2 21607538

[B43] Schwachmeyer V. Damm P. Bender A. Dymke J. Graichen F. Bergmann G. (2013). *In Vivo* hip Joint Loading during post-operative Physiotherapeutic Exercises. PLoS One 8 (10), e77807. 10.1371/journal.pone.0077807 24204977 PMC3812157

[B44] Swanson E. A. Schmalzried T. P. Dorey F. J. (2009). Activity Recommendations after Total Hip and Knee Arthroplasty: a Survey of the American Association for Hip and Knee Surgeons. J. Arthroplasty 24 (6 Suppl. l), 120–126. 10.1016/j.arth.2009.05.014 19698910

[B45] Tirosh O. Sparrow W. A. (2005). Age and Walking Speed Effects on Muscle Recruitment in Gait Termination. Gait & Posture 21 (3), 279–288. 10.1016/j.gaitpost.2004.03.002 15760743

[B46] Trudelle-Jackson E. Smith S. S. (2004). Effects of a Late-phase Exercise Program after Total Hip Arthroplasty: a Randomized Controlled Trial. Arch. Phys. Med. Rehabil. 85 (7), 1056–1062. 10.1016/j.apmr.2003.11.022 15241750

[B47] Unlu E. Eksioglu E. Aydog E. Aydoð S. T. Atay G. (2007). The Effect of Exercise on Hip Muscle Strength, Gait Speed and Cadence in Patients with Total Hip Arthroplasty: a Randomized Controlled Study. Clin. Rehabil. 21 (8), 706–711. 10.1177/0269215507077302 17846070

[B48] Viceconti M. Muccini R. Bernakiewicz M. Baleani M. Cristofolini L. (2000). Large-sliding Contact Elements Accurately Predict Levels of Bone-Implant Micromotion Relevant to Osseointegration. J. Biomech. 33 (12), 1611–1618. 10.1016/s0021-9290(00)00140-8 11006385

[B49] Vogel L. A. Carotenuto G. Basti J. J. Levine W. N. (2011). Physical Activity after Total Joint Arthroplasty. Sports Health 3 (5), 441–450. 10.1177/1941738111415826 23016041 PMC3445215

[B50] Vu-Han T. Hardt S. Ascherl R. Gwinner C. Perka C. (2020). Recommendations for Return to Sports after Total Hip Arthroplasty Are Becoming Less Restrictive as Implants Improve. Arch. Orthop. Trauma Surg. 141 (3), 497–507. 10.1007/s00402-020-03691-1 33258998 PMC7899958

[B51] Wu G. Siegler S. Allard P. Kirtley C. Leardini A. Rosenbaum D. (2002). ISB Recommendation on Definitions of Joint Coordinate System of Various Joints for the Reporting of Human Joint Motion-Part I: Ankle, Hip, and Spine. J. Biomech. 35 (4), 543–548. 10.1016/s0021-9290(01)00222-6 11934426

