# Supplementary material for: Is Training With Gym Machines Safe After Hip Arthroplasty?—An In Vivo Load Investigation
Source: Front Bioeng Biotechnol. 2022 Mar 24;10:857682. doi: 10.3389/fbioe.2022.857682 (PMC8989469; doi:10.3389/fbioe.2022.857682)
Supplement: Supplementary file 1 [file DataSheet1.docx]

| **F_res_** |  |  |  |  |  |  |  |
| --- | --- | --- | --- | --- | --- | --- | --- |
|  | H2R | H3L | H4L | H5L | H6R | H7R | H8L |
| Individual Median Walking | 275 | 263 | 298 | 334 | 326 | 323 | 300 |
| Leg Curl 20kg | 89 | 121 | 117 | 126 | 72 | 122 | 100 |
| Leg Curl 30kg | - | 159 | 160 | 212 | 103 | 161 | 149 |
| Leg Curl 40kg | 176 | - | 228 | 255 | 166 | 228 | 183 |
| Leg Extension 20kg | 75 | 210 | 68 | 120 | 60 | 82 | 91 |
| Leg Extension 30kg | - | 222 | 82 | 172 | 75 | 119 | 113 |
| Leg Extension 40kg | 112 | - | 112 | 231 | 92 | 133 | 137 |
| Leg Press, backrest 10°, 50%BW | 96 | 161 | 88 | 197 | 121 | 149 | 190 |
| Leg Press, backrest 10°, 75%BW | 135 | - | 125 | 307 | 162 | 210 | 240 |
| Leg Press, backrest 10°, 100%BW | 163 | - | 194 | **438** | 210 | 260 | 293 |
| Leg Press, backrest 30°, 50%BW | - | - | 83 | 249 | 124 | 130 | 212 |
| Leg Press, backrest 30°, 75%BW | - | - | 108 | **361** | 160 | 192 | 229 |
| Leg Press, backrest 30°, 100%BW | 274 | - | 137 | - | 197 | 250 | 295 |
| Leg Press, backrest 60°, 50%BW | 129 | 138 | 92 | 198 | 105 | 159 | 200 |
| Leg Press, backrest 60°, 75%BW | 172 | - | 120 | 260 | 143 | 212 | 237 |
| Leg Press, backrest 60°, 100%BW | 238 | - | 173 | - | 163 | 270 | 291 |
| Rope Pull, ipsilateral performed, Adduction | - | - | 150 | 85 | 108 | 164 | 94 |
| Rope Pull, ipsilateral performed, Abduction | - | - | 171 | 130 | 186 | 220 | 126 |
| Rope Pull, ipsilateral performed, Flexion | - | - | 160 | 162 | 179 | 192 | 179 |
| Rope Pull, ipsilateral performed, Extension | - | - | 224 | 271 | 192 | 221 | 144 |
| Rope Pull, contralateral performed, Adduction | - | - | 252 | 304 | 260 | 288 | 294 |
| Rope Pull, contralateral performed, Abduction | - | - | **376** | **371** | 310 | **327** | **347** |
| Rope Pull, contralateral performed, Flexion | - | - | **317** | **385** | 308 | **367** | **310** |
| Rope Pull, contralateral performed, Extension | - | - | **300** | 301 | 310 | 305 | **300** |

**Supplement Table 1.** **Individual median for each investigated participant for the reference activity walking and each investigated activity depicting the resultant force F_res_,** with the results given as median values in %BW; **bold** – individual determined median value of the exercise is exceeding the individual median of walking of this participant. Ipsilateral and contralateral regarding the standing leg does indicate either the implanted instrumented hip prothesis (ipsilateral) or the not operated side (contralateral). Rope pull exercises performed with ipsilateral side, standing on the contralateral leg, referred to as ipsilateral. Rope pull exercises performed with contralateral side, standing on the ipsilateral leg, referred to as contralateral.

| **M_bend_** |  |  |  |  |  |  |  |
| --- | --- | --- | --- | --- | --- | --- | --- |
|  | H2R | H3L | H4L | H5L | H6R | H7R | H8L |
| Individual Median Walking | 4.09 | 4.31 | 4.22 | 4.36 | 3.71 | 5.57 | 3.51 |
| Leg Curl 20kg | 1.06 | 1.54 | 1.91 | 1.66 | 0.76 | 1.85 | 1.25 |
| Leg Curl 30kg | - | 2.88 | 2.82 | 2.98 | 1.26 | 2.65 | 2.11 |
| Leg Curl 40kg | 2.27 | - | 3.91 | 3.56 | 1.80 | 3.97 | 2.68 |
| Leg Extension 20kg | 1.06 | 2.19 | 0.72 | 1.83 | 0.22 | 1.15 | 0.55 |
| Leg Extension 30kg | - | 2.44 | 0.90 | 2.37 | 0.33 | 1.56 | 0.71 |
| Leg Extension 40kg | 1.67 | - | 1.53 | 3.05 | 0.53 | 1.83 | 0.95 |
| Leg Press, backrest 10°, 50%BW | 1.04 | 2.66 | 1.18 | 2.25 | 1.45 | 2.11 | 1.91 |
| Leg Press, backrest 10°, 75%BW | 1.55 | - | 1.79 | 3.76 | 2.02 | 2.86 | 2.67 |
| Leg Press, backrest 10°, 100%BW | 1.84 | - | 2.82 | **5.31** | 2.61 | 3.63 | **3.67** |
| Leg Press, backrest 30°, 50%BW | - | - | 1.16 | 2.45 | 1.88 | 1.63 | 2.01 |
| Leg Press, backrest 30°, 75%BW | - | - | 1.57 | 3.76 | 2.46 | 2.73 | 2.40 |
| Leg Press, backrest 30°, 100%BW | 2.52 | - | 2.07 | - | 3.08 | 3.64 | 3.52 |
| Leg Press, backrest 60°, 50%BW | 1.35 | 2.32 | 1.28 | 2.73 | 1.49 | 2.06 | 1.83 |
| Leg Press, backrest 60°, 75%BW | 1.87 | - | 1.69 | 4.00 | 2.25 | 3.45 | 2.28 |
| Leg Press, backrest 60°, 100%BW | 2.54 | - | 2.26 | - | 2.52 | 4.66 | 3.10 |
| Rope Pull, ipsilateral performed, Adduction | - | - | 1.09 | 0.80 | 0.38 | 1.20 | 0.49 |
| Rope Pull, ipsilateral performed, Abduction | - | - | 1.89 | 1.26 | 1.68 | 3.34 | 1.08 |
| Rope Pull, ipsilateral performed, Flexion | - | - | 1.84 | 2.10 | 1.57 | 2.45 | 1.43 |
| Rope Pull, ipsilateral performed, Extension | - | - | 3.11 | 3.76 | 2.56 | 3.42 | 1.84 |
| Rope Pull, contralateral performed, Adduction | - | - | 3.51 | 3.71 | 3.14 | 4.84 | **4.08** |
| Rope Pull, contralateral performed, Abduction | - | - | **4.63** | 4.24 | **4.08** | 5.01 | **3.65** |
| Rope Pull, contralateral performed, Flexion | - | - | **4.81** | **5.06** | **4.68** | **5.76** | **3.99** |
| Rope Pull, contralateral performed, Extension | - | - | **4.85** | 3.75 | **4.34** | 4.75 | **4.15** |

**Supplement Table 2.** **Individual median for each investigated participant for the reference activity walking and each investigated activity depicting the bending moment M_bend_** with the results given as median values in %BWm; **bold** – individual determined median value of the exercise is exceeding the individual median of walking of this participant. Ipsilateral and contralateral regarding the standing leg does indicate either the implanted instrumented hip prothesis (ipsilateral) or the not operated side (contralateral). Rope pull exercises performed with ipsilateral side, standing on the contralateral leg, referred to as ipsilateral. Rope pull exercises performed with contralateral side, standing on the ipsilateral leg, referred to as contralateral.

| **M_tors_** |  |  |  |  |  |  |  |
| --- | --- | --- | --- | --- | --- | --- | --- |
|  | H2R | H3L | H4L | H5L | H6R | H7R | H8L |
| Individual Median Walking | 2.48 | 2.47 | 3.01 | 2.59 | 3.09 | 2.88 | 2.41 |
| Leg Curl 20kg | 0.99 | 1.02 | 1.11 | 0.84 | 0.52 | 0.69 | 1.18 |
| Leg Curl 30kg | - | 2.37 | 1.35 | 1.46 | 0.77 | 1.19 | 1.58 |
| Leg Curl 40kg | 1.88 | - | 2.18 | 1.40 | 2.03 | 2.29 | 2.25 |
| Leg Extension 20kg | 1.30 | **2.65** | 1.19 | 1.77 | 0.96 | 1.52 | 1.74 |
| Leg Extension 30kg | - | **2.93** | 1.50 | 2.32 | 1.30 | 2.20 | 2.19 |
| Leg Extension 40kg | 2.16 | - | 1.95 | **3.08** | 1.55 | 2.50 | **2.69** |
| Leg Press, backrest 10°, 50%BW | 0.30 | 1.37 | 1.23 | 1.53 | 1.08 | 1.13 | 0.97 |
| Leg Press, backrest 10°, 75%BW | 0.71 | 1.48 | 2.14 | 1.49 | 1.81 | 1.81 | 0.86 |
| Leg Press, backrest 10°, 100%BW | 0.78 | - | 1.92 | **3.01** | 1.81 | 2.28 | **2.76** |
| Leg Press, backrest 30°, 50%BW | - | - | 1.18 | 1.83 | 1.69 | 0.96 | 1.12 |
| Leg Press, backrest 30°, 75%BW | - | - | 1.33 | 2.31 | 1.40 | 1.56 | 1.79 |
| Leg Press, backrest 30°, 100%BW | 0.86 | - | 1.62 | - | 1.68 | 2.10 | **2.59** |
| Leg Press, backrest 60°, 50%BW | 0.44 | 1.45 | 1.23 | 1.95 | 0.72 | 1.02 | 1.37 |
| Leg Press, backrest 60°, 75%BW | 0.62 | - | 1.65 | 2.02 | 1.24 | 1.25 | 1.43 |
| Leg Press, backrest 60°, 100%BW | 0.91 | - | 2.33 | - | 1.68 | 1.64 | 2.30 |
| Rope Pull, ipsilateral performed, Adduction | - | - | 0.84 | 0.41 | 0.22 | 0.88 | 0.29 |
| Rope Pull, ipsilateral performed, Abduction | - | - | 1.33 | 1.14 | 1.39 | **3.08** | 0.68 |
| Rope Pull, ipsilateral performed, Flexion | - | - | 2.07 | 1.78 | 2.09 | 2.77 | **2.87** |
| Rope Pull, ipsilateral performed, Extension | - | - | 1.27 | 1.29 | 1.05 | 1.35 | 0.84 |
| Rope Pull, contralateral performed, Adduction | - | - | 0.81 | 1.19 | 0.58 | 2.03 | 0.50 |
| Rope Pull, contralateral performed, Abduction | - | - | 2.98 | 1.96 | 2.21 | 2.60 | 1.22 |
| Rope Pull, contralateral performed, Flexion | - | - | 2.21 | 1.95 | 2.13 | **3.03** | 1.78 |
| Rope Pull, contralateral performed, Extension | - | - | 0.49 | 0.76 | 2.07 | 1.54 | 0.81 |

**Supplement Table 3.** **Individual median for each investigated participant for the reference activity walking and each investigated activity depicting the torsion torque M_tors_** with the results given as median values in %BWm; **bold** – individual determined median value of the exercise is exceeding the individual median of walking of this participant. Ipsilateral and contralateral regarding the standing leg does indicate either the implanted instrumented hip prothesis (ipsilateral) or the not operated side (contralateral). Rope pull exercises performed with ipsilateral side, standing on the contralateral leg, referred to as ipsilateral. Rope pull exercises performed with contralateral side, standing on the ipsilateral leg, referred to as contralateral.
